# Adherence to the American Cancer Society guidelines on nutrition and physical activity for cancer survivors and biomarkers of inflammation among breast cancer survivors

**DOI:** 10.4178/epih.e2024026

**Published:** 2024-01-25

**Authors:** Minji Kang, Sihan Song, Hyun Jeong Cho, Zisun Kim, Hyun Jo Youn, Jihyoung Cho, Jun Won Min, Yoo Seok Kim, Sang-Woon Choi, Jung Eun Lee

**Affiliations:** 1 Department of Food and Nutrition, Duksung Women’s University, Seoul, Korea; 2 Department of Food and Nutrition, Seoul National University, Seoul, Korea; 3 K-BIO KIURI Center, Seoul National University, Seoul, Korea; 4 Department of Surgery, Soonchunhyang University Bucheon Hospital, Bucheon, Korea; 5 Department of Surgery, Jeonbuk National University Medical School, Jeonju, Korea; 6 Department of Surgery, Keimyung University School of Medicine, Daegu, Korea; 7 Department of Surgery, Dankook University College of Medicine, Cheonan, Korea; 8 Department of Surgery, Chosun University College of Medicine, Gwangju, Korea; 9 Chaum Life Center, CHA University, Seoul, Korea; 10 Department of Nutrition, School of Public Health and Health Sciences, University of Massachusetts Amherst, Amherst, MA, USA; 11 Research Institute of Human Ecology, Seoul National University, Seoul, Korea

**Keywords:** ACS guidelines for cancer survivors, Healthy lifestyle, Inflammatory marker, Breast cancer survivor

## Abstract

**OBJECTIVES:**

This study investigated whether adherence to the overall lifestyle recommendations in the American Cancer Society (ACS) guidelines on nutrition and physical activity for cancer survivors was associated with inflammation in breast cancer survivors.

**METHODS:**

The study included 409 women who had undergone breast cancer surgery at least 1 year before enrollment. A generalized linear model was used to estimate the least square means and 95% confidence intervals of plasma levels of inflammatory markers according to lifestyle factors defined in terms of adherence to the ACS guidelines.

**RESULTS:**

Higher overall adherence scores were associated with lower levels of high-sensitivity C-reactive protein (hs-CRP) (p for trend=0.015) and higher levels of adiponectin (p for trend=0.009). Similar significant associations of hs-CRP (p for trend= 0.004) and adiponectin (p for trend=0.010) levels were observed with the score for the body mass index (BMI) component of the adherence score. A higher diet component score was associated with a higher adiponectin level (p for trend=0.020), but there was no significant association for the physical activity component score.

**CONCLUSIONS:**

The present study’s findings suggest that maintaining a healthy lifestyle according to the ACS guidelines was associated with beneficial effects on inflammatory marker levels, especially hs-CRP and adiponectin, among breast cancer survivors. Among the 3 components of lifestyle guidelines, the BMI component exhibited the most similar tendency to the overall adherence score in relation to inflammatory indicators. Further prospective and intervention studies are needed to investigate longitudinal associations between lifestyle factors and inflammatory markers among breast cancer survivors.

## GRAPHICAL ABSTRACT

[Fig f1-epih-46-e2024026]

## Key Message

• Maintaining a healthy lifestyle according to the American Cancer Society (ACS) Guidelines on Nutrition and Physical Activity for Cancer Survivors was associated with favorable levels of inflammatory markers, especially in hs-CRP and adiponectin among breast cancer survivors.

• Maintaining an adequate BMI of 18.5 to 23 kg/m^2^ was associated with lower levels of hs-CRP and higher adiponectin levels.

• In addition, a healthy diet — indicating a diet high in fruits, vegetables, and whole grains, and low in red and processed meats — was associated with higher adiponectin levels.

## INTRODUCTION

Breast cancer was the most frequently diagnosed cancer among women worldwide in 2018, accounting for 24.2% of all cases and an age-standardized rate of 46.3 per 100,000 [1]. It was also the leading cause of cancer death in women, representing 15% of all cancer deaths with an age-standardized rate of 13.0 per 100,000 [1]. In Korea, the incidence of breast cancer has been continuously rising since 1999, reaching an age-standardized rate of 55.6 per 100,000 women in 2017 [2]. Moreover, the 5-year relative survival rate for breast cancer patients diagnosed between 2013 and 2017 was 93.2%, a significant increase from the 79.2% recorded between 1993 and 1995 [2]. Therefore, there is increasing interest in dietary and lifestyle factors associated with improved survival rates and quality of life among breast cancer survivors.

Several recommendations have noted the importance of lifestyle choices for improving survival rates among breast cancer survivors and reducing the risk of secondary disease diagnosis [3,4]. For instance, the World Cancer Research Fund (WCRF) Continuous Update Project report on breast cancer survivors indicates that there is some evidence of a correlation between improved survival post-breast cancer and lifestyle factors. These factors include maintaining a healthy weight, engaging in regular physical activity, consuming fiber-rich foods, and reducing the intake of total and particularly saturated fats [4]. Guidelines aimed at lifestyle management to improve the overall health of individuals after breast cancer diagnosis are continually being refined. The American Cancer Society (ACS), which provides one of the most widely referenced guidelines, highlights the role of weight management, physical activity, and diet for cancer survivors [3]. Specifically, the ACS guidelines on nutrition and physical activity for cancer survivors emphasize the need for breast cancer survivors to maintain a healthy weight by consuming a balanced diet rich in vegetables, fruits, and whole grains, and low in red and processed meats [3]. Additionally, engaging in regular exercise is recommended to lower the risks of breast cancer recurrence, cardiovascular disease, and common comorbidities [3].

Inflammation is a critical component of tumorigenesis, tumor progression, and tumor metastasis [5,6]. Chronic inflammation may be associated with increased risks of cardiovascular disease [7,8] and type 2 diabetes [9,10], as well as breast cancer recurrence and mortality [11-13]. For example, a study of 734 disease-free breast cancer survivors in the Health, Eating, Activity, and Lifestyle (HEAL) study indicated that elevated levels of inflammatory markers, including C-reactive protein (CRP), were associated with reduced overall survival over a follow-up of approximately 4.1 years, even after adjustment for age, tumor stage, race, and body mass index (BMI; p for trend= 0.002) [11].

It is widely accepted that a healthy diet and physical activity can significantly reduce inflammation [14-17]. However, there have been few studies examining the impact of overall lifestyle factors, guided by nutrition and physical activity recommendations for cancer survivors, on inflammation in breast cancer survivors. This cross-sectional study evaluated the associations between overall lifestyle factors, as determined by adherence to the ACS guidelines on nutrition and physical activity for cancer survivors, and levels of inflammatory markers among breast cancer survivors.

## MATERIALS AND METHODS

### Study participants

A total of 520 women who had been diagnosed with invasive primary breast cancer stages I-III, according to the American Joint Committee on Cancer (AJCC) staging system, were recruited from 5 hospitals in Korea between March 2015 and June 2019. All these women had undergone breast cancer surgery at least 1 year before enrollment. Clinical information, such as cancer stage, hormone receptor status, and time since surgery, was collected from the medical records at each hospital. We excluded participants diagnosed with other cancers before enrollment (n = 9) and those lacking information on BMI (n = 5), dietary intake (n = 79), or inflammatory markers (n = 7). In addition, participants who disclosed implausible energy intake (±3 standard deviations [SDs] from the mean of the log-transformed energy intake, n = 10) were also excluded. Therefore, 409 women were eligible for inclusion in this study.

### Adherence to the American Cancer Society guidelines on nutrition and physical activity for cancer survivors

Adherence scores were assessed using the ACS guidelines on nutrition and physical activity for cancer prevention [3]. The total adherence score ranged from 3 to 12 and consisted of 3 components, each scored from 1 (worst) to 4 (best): achieving and maintaining a healthy weight; engaging in regular physical activity; and consuming a diet high in fruits, vegetables, and whole grains and low in red and processed meat [3,18].

BMI (kg/m^2^) was calculated by dividing weight (kg) by the square of height (m^2^) measured at enrollment. The lowest score for the BMI component was assigned to < 18.5 or ≥ 30.0 kg/m^2^, and participants with BMI 25.0-30.0 kg/m^2^, 23.0-25.0 kg/m^2^, and 18.5-23.0 kg/m^2^ received scores of 2, 3, and 4, respectively, based on the Asia-Pacific classification for obesity [19].

As a measure of physical activity, participants completed a questionnaire describing the type, duration, and frequency of exercise they regularly engaged in. For each exercise type, we used the formula for metabolic equivalents of task (MET) to calculate the MET-hr/wk [20]. The total number of MET-hr/wk was derived by adding the MET-hr/wk values calculated for each exercise type. Participants were classified into 4 groups according to their MET-hr/wk of total physical activity. The lowest quartile received 1 point, while the highest quartile received 4 points.

Dietary patterns were assessed from dietary data collected based on either 3-day dietary records (3DRs) for 217 women or a food frequency questionnaire (FFQ) for 192 women. The Computer-Aided Nutritional Analysis Program (CAN-pro) version 4.0, developed by the Korean Nutrition Society, was used to calculate the average dietary intake from 3DRs. The FFQ was developed for Korean breast cancer survivors and included 126 food and beverage items, validated based on dietary records [21,22]. In a validation study, the correlation coefficients for validity ranged from 0.20 (riboflavin) to 0.53 (calcium), with a median of 0.37 [22]. The FFQ included a variety of fruits and vegetables, such as grapes, persimmons, peaches, apples, strawberries, tangerines, bananas, kiwis, spinach, perilla leaves, bean sprouts, mung bean sprouts, cabbage, lettuce, cucumber, radish, green onions, garlic, kimchi, and pickles. Whole grains, such as multigrain rice and bread were also included, while the category of red and processed meats consisted of pork, beef, and ham. Based on their daily intake (g/day), the participants were classified into 4 groups according to their consumption of the following 3 food groups: fruits and vegetables, whole grains, and red and processed meat. More specifically, the diet score was calculated based on the amount of fruit and vegetable intake, with 1 being the lowest quartile (< 362.5 g from all participants [< 360.2 g based on 3DRs, < 362.4 g based on FFQ]) and 4 being the highest quartile (> 833.0 g from all participants [> 833.0 g based on 3DRs, > 834.1 g based on FFQ]). Similarly, the score was calculated based on the amount of whole grain intake, with 1 being the lowest quartile (< 44.1 g from all participants [< 43.3 g based on 3DRs, < 44.1 g based on FFQ]) and 4 being the highest quartile (> 150 g from all participants [> 150.0 g based on 3DRs, > 184.6 g based on FFQ]). For red and processed meat, the lowest quartile (< 12.3 g from all participants [< 12.3 g based on 3DRs, < 11.5 g based on FFQ]) received the highest score, and the highest quartile (> 90.4 g from all participants [> 90.5 g based on 3DRs, >92.3 g based on FFQ]) received the lowest score. The scores of the 3 food groups were summed and classified into 4 groups based on the quartiles of the calculated score (1 for the lowest quartile to 4 for the highest quartile).

### Inflammatory markers

Non-fasting blood samples were collected at each hospital during interviews with participants. The plasma samples were frozen and sent to a central laboratory (Seegene, Seoul, Korea). Markers of inflammation were measured through the assessment of plasma concentrations of high-sensitivity CRP (hs-CRP), interleukin (IL)-6, IL-8, tumor necrosis factor-α (TNF-α), and adiponectin.

The level of plasma hs-CRP was measured by a particle-enhanced immunoturbidimetric assay, which was conducted using a Cobas 8000 C702 high-throughput immunochemistry module (Roche Diagnostics, Mannheim, Germany). The lowest level that could be detected for hs-CRP was 0.15 mg/L, and the inter-assay coefficient of variation (CV) was 2.3%. Samples with an hs-CRP value below the limit of detection were assigned a plasma value of half the detection limit (16.4%). Plasma concentrations of IL-6, IL-8, and TNF-α were measured by multiplex immunoassay at the Life is the Art of Science (LAS) Laboratory in Gimpo, Korea. We collected data from blood samples between 2015 and 2017 using a Luminex 200 instrument (Luminex, Austin, TX, USA). We analyzed these samples using Bio-plex Manager 6.0 (Bio-Rad, Hercules, CA, USA). For blood samples collected after 2018, we used a MAGPIX instrument (Luminex) and analyzed them using MasterPlex QT 2010 (MiraiBio, Hitachi, CA, USA). The intra-assay and inter-assay CVs for IL-6 were 2.2% and 3.0%, respectively. For IL-8, the CVs were 3.2% and 2.8%, and for TNF-α, they were 3.5% and 3.0%, respectively. The level of plasma adiponectin was measured using an enzyme-linked immunosorbent assay with a microplate reader (VersaMax; Molecular Devices, Downingtown, PA, USA) at CHA Bio Complex (CHA University, Seongnam, Korea). The intra-assay CVs ranged from 4.2% to 5.0%. The IL-6, IL-8, TNF-α, and adiponectin levels were replaced with 0 for samples in which they were outside the measurement range (5.4, 0.0, 2.7, and 0.0% of samples, respectively).

### Statistical analysis

A generalized linear model was used to estimate the least-squares (LS)-means and 95% confidence intervals (CIs) of plasma inflammatory marker levels according to lifestyle factors measured in terms of adherence to the ACS guidelines on nutrition and physical activity for cancer survivors. The normality of the data was improved by the natural log transformation of plasma inflammatory marker levels. We adjusted for the inflammatory markers based on the residual method to account for any differences between the 2 measurement time points. The total ACS guideline scores were divided into quartiles, with each of the 3 components (i.e., BMI, physical activity, and diet) divided into 4 groups based on scores of 1 to 4. Analyses were adjusted for the following socio-demographic and health-related variables: age (years; continuous), energy intake (log-transformed energy intake, kcal/day; continuous), education level (elementary school or below, middle school, high school, and college or above), marital status (married or cohabiting, unmarried or divorced or widowed), menopausal status at diagnosis (premenopausal or postmenopausal), AJCC stage at diagnosis (I, II, or III), time since surgery (1-2, 2-5, or ≥ 5 years), estrogen receptor (ER) status (negative or positive), history of chronic diseases (no or yes), smoking status (never, ever), alcohol intake (nondrinker, < 1, ≥ 1 drink/day), and dietary supplement use (yes or no). In addition, to account for measurements at multiple medical centers, we included the medical center (5 centers) as a covariate in the model. We conducted subgroup analyses to determine the association between inflammatory markers and adherence scores to the ACS guidelines by age at enrollment, AJCC breast cancer stage, ER status, menopausal status at diagnosis, and time since surgery. We also examined whether replacing inflammatory marker values changed the association with ACS guidelines. Data were analyzed using SAS version 9.4 (SAS Institute Inc., Cary, NC, USA), and differences were considered significant at p-value < 0.05.

### Ethics statement

Written informed consent was obtained from all participants, and all procedures were approved by the institutional review boards of the following 5 participating hospitals: Chosun University Hospital (CHOSUN2018-06-004-001), Dankook University Hospital (DKUH2020-10-003-004), Jeonbuk National University Hospital (CUH2018-02-004-004), Keimyung University Dongsan Medical Center (DSMC2015-03-026-023), and Soonchunhyang University Hospital (SCHBC2014-12-004-001).

## RESULTS

Table 1 presents the characteristics of the study participants based on their adherence scores. The mean± SD values for age, BMI, physical activity, and energy intake were 52.1± 8.2 years, 23.4± 3.1 kg/m^2^, 33.3± 38.7 MET-hr/wk, and 1,762.8± 567.5 kcal/day, respectively. Among the 409 participants included in the analysis, 78.7% were married or cohabiting, 65.5% were premenopausal at diagnosis, and 64.3% used dietary supplements. Most participants were non-smokers (91.9%) and non-drinkers (80.0%) at enrollment. Nearly half of the participants in our study were diagnosed with stage I breast cancer (48.9%), and 80.2% were enrolled within 5 years of breast cancer surgery.

The plasma levels of inflammatory markers among breast cancer survivors are presented in Table 2, categorized by quartiles of overall adherence scores. Higher adherence scores were associated with lower levels of hs-CRP (p for trend= 0.015). The LS-means (95% CIs) of the lowest and the highest quartiles of adherence scores were 0.71 mg/L (95% CI, 0.48 to 0.99) and 0.52 mg/L (95% CI, 0.30 to 0.78), respectively. Higher adherence scores were also associated with higher levels of adiponectin (p for trend= 0.009). The LSmeans (95% CIs) of the lowest and highest quartiles of adherence scores were 7.94 μg/mL (95% CI, 6.17 to 10.14) and 10.30 μg/mL (95% CI, 7.96 to 13.24), respectively. Adherence scores were not significantly associated with IL-6, IL-8, or TNF-α levels. Similar results were observed with the exclusion of participants with replaced values for each inflammatory marker (Supplementary Material 1).

We also analyzed the associations between inflammatory markers and scores for each of the 3 components (i.e., BMI, physical activity, and diet) (Table 3). A higher BMI component score (a score of 4) indicated that the recommended BMI was met, and higher BMI component scores were associated with lower levels of hs-CRP (p for trend= 0.004) and higher levels of adiponectin (p for trend= 0.010). In addition, higher diet component scores (dietary pattern high in fruits, vegetables, and whole grains and low in red and processed meat) were associated with higher adiponectin levels (p for trend= 0.020). There were no significant associations between physical activity score and inflammatory markers. When we additionally adjusted for the 2 other components in models for each component (i.e., BMI, physical activity, and diet), similar results were observed (Supplementary Material 2).

We examined whether the associations between the adherence scores and inflammatory markers were modified by age at enrollment, menopausal status at diagnosis, AJCC breast cancer stage, time since surgery, or ER status (Table 4). The subgroup analyses showed that the inverse association between the adherence scores and the levels of hs-CRP slightly weakened in women who were younger than 50 years, premenopausal, AJCC stage II-III, had undergone surgery more than 2 years ago, or had ER-positive status, and the associations were no longer statistically significant. However, the test for interaction did not show statistically significant differences in the associations across the subgroups (p-values for interaction > 0.22). The positive association between adherence scores and adiponectin levels was only significant in women who were premenopausal, AJCC stage II-III, or had undergone surgery more than 2 years ago. However, there were no significant interactions observed in the associations across subgroups (p-values for interaction > 0.29). Adherence scores did not show significant associations with IL-6, IL-8, or TNF-α levels, except for IL-8, in women under 50 years old.

## DISCUSSION

The present study examined associations between lifestyle factors assessed in terms of adherence to the ACS guidelines on nutrition and physical activity for cancer survivors and plasma levels of inflammatory markers among breast cancer survivors. Higher adherence scores and BMI component scores were associated with lower levels of hs-CRP and higher levels of adiponectin. In addition, a higher diet component score—indicating a diet high in fruits, vegetables, and whole grains and low in red and processed meat—was associated with higher adiponectin levels. Adherence scores were not significantly associated with IL-6, IL-8, or TNF-α levels.

Only a few studies have investigated inflammatory indicators among cancer survivors who follow the overall lifestyle guidelines, especially the ACS guidelines on nutrition and physical activity for cancer survivors. Additionally, some studies have examined biomarkers among women without any disease or with breast cancer based on adherence to the World Cancer Research Fund/American Institute for Cancer Research (WCRF/AICR) guidelines. A study using data from 11,342 disease-free women in the Nurses’ Health Study showed a significant trend of lower (higher for adiponectin) biomarker concentrations with greater adherence to the cancer prevention recommendations in the WCRF/AICR 2007 report [23]. A study of 275 healthy premenopausal women showed that following the recommended guidelines was associated with lower levels of biomarkers associated with oxidative stress and inflammation [24]. One cross-sectional study examined associations between the prevalence of metabolic syndrome and adherence to the recommendations for cancer prevention among patients with breast cancer [25]. Although there have been few studies regarding the compliance of breast cancer survivors to the ACS guidelines, numerous studies of breast cancer survivors examined BMI, physical activity, and diet, which are included as components of the ACS guidelines for cancer survivors.

There is accumulating evidence that overweight and obesity are risk factors for poorer prognosis and a variety of undesirable outcomes, including recurrence, comorbidities, and overall mortality, in cancer survivors [3,26]. A meta-analysis based on prospective studies, which considered the relationship of excess body weight to second primary cancer risk after breast cancer, showed that obesity was associated with an increased relative risk (RR) of contralateral breast (RR, 1.37; 95% CI, 1.20 to 1.57), breast (RR, 1.40; 95% CI, 1.24 to 1.58), endometrial (RR, 1.96; 95% CI, 1.43 to 2.70), and colorectal (RR, 1.89; 95% CI, 1.28 to 2.79) secondary cancers [26]. In addition, higher levels of adiponectin, which is inversely correlated with BMI [27], were associated with more prolonged breast cancer survival (hazard ratio, 0.39; 95% CI, 0.15 to 0.95) among 527 women in the HEAL study [28]. Although there have been relatively few reports of associations between BMI and inflammatory markers among breast cancer survivors, a recent study showed that a higher BMI category, such as ≥ 25.0 kg/m^2^, was significantly associated with higher CRP levels among 201 breast cancer survivors [29]. In comparison with this previous study, our study population was relatively lean. In our study, BMI component scores (BMI between 18.5 and 23.0 kg/m^2^) were significantly associated with lower concentrations of hs-CRP and higher concentrations of adiponectin, in addition to the overall score for adherence to the ACS guidelines. Among the 3 components of the ACS guidelines—namely, BMI, physical activity, and dietary patterns—the relationship between BMI component scores and inflammatory indicators was found to be the most similar to that of the overall score of adherence with the ACS guidelines and inflammatory indicators. Considering that BMI is an indicator of long-term eating status, it was concluded that the influence of BMI could explain many of the 3 components. Our findings support universal recommendations for cancer survivors to achieve and maintain a healthy weight.

In this cross-sectional study, we did not find any significant associations between physical activity scores and inflammatory markers. However, we observed a marked difference in MET-hr/wk between the group with the highest physical activity score (68.9 MET-hr/wk) and the group with the lowest physical activity score (12.1 MET-hr/wk). To better understand this inconsistency, further investigation is required, such as analyzing the types and intensity of activities that contribute to the physical activity score or examining a wider range of inflammatory markers. A recent meta-analysis of exercise training and pro-inflammatory and anti-inflammatory markers among cancer survivors emphasized exercise training for cancer survivors and reported that combined aerobic and resistance training had the greatest effect (standardized mean difference, -0.3; 95% CI, -0.5 to -1.9; p< 0.001) [30]. Furthermore, several interventions have evaluated the influence of physical activity on inflammatory markers in breast cancer survivors [31,32]. For example, a randomized controlled trial was conducted among 200 breast cancer survivors assigned to either 12 weeks of 90-minute yoga classes twice per week or a control group. In that previous study, no significant between-group differences were observed immediately following treatment, but by 3 months post-treatment, the yoga group had significantly reduced levels of cytokines, including IL-6, TNF-α, and IL-1β [33]. In another study, a randomized controlled trial examined the effects of exercise on changes in inflammatory profile in postmenopausal breast cancer survivors. No significant difference was found between women randomized to 6 months of aerobic exercise or a control group receiving standard care; however, a significant reduction in IL-6 was found among those in the exercise group who reached 80% of the intervention goal compared with those who did not [34].

A higher score for the diet component—indicating a diet high in fruits, vegetables, and whole grains and low in red and processed meat—was significantly associated with higher levels of adiponectin after adjustment for potential covariates, but there were no such differences in serum concentrations of other inflammatory markers. The result of the association between diet components and adiponectin is consistent with the fact that a plant-based diet, which is high in fruits, vegetables, and whole grains, as well as a low-energy diet and a diet rich in dietary fiber contained in fruit, vegetables, and whole grains, has a beneficial effect on improving adiponectin concentration [35]. Moreover, diet can influence inflammatory indicators both indirectly and directly. For example, BMI status reflects long-term nutritional intake; thus, dietary factors can indirectly affect associations, such as excessive BMI being associated with higher levels of inflammation [36]. Furthermore, specific dietary ingredients, such as fiber, magnesium, carotenoids, and flavonoids found in a diet rich in fruits, vegetables, and grains, can have a direct effect on inflammation [37]. Similar to our study, several cross-sectional analyses have reported the anti-inflammatory effects of a healthy diet as measured by diet quality indices, including the Healthy Eating Index and the Mediterranean Diet Score, in breast cancer survivors [14-16]. For example, a better-quality diet, such as one high in fruits and vegetables and low in saturated fat, sodium, and added sugar, seemed to be associated with lower levels of chronic inflammation among breast cancer survivors [14]. Furthermore, a meta-analysis of cohort studies reported that adherence to diet quality indices and a prudent/healthy dietary pattern was associated with reduced risk of overall mortality (diet quality indices: RR, 0.74; 95% CI, 0.60 to 0.90; prudent/healthy dietary pattern: RR, 0.76; 95% CI, 0.60 to 0.95) among breast cancer survivors [38].

Additionally, we conducted a subgroup analysis to investigate whether the association between the overall adherence score and inflammatory markers varied by age, menopausal status at diagnosis, breast cancer stage, duration of surgery, and ER status in breast cancer survivors. Our results showed that the overall adherence score had a significant inverse association with hs-CRP among all participants. However, this relationship remained significant only in several subgroups, including women aged 50 years or older, women who were postmenopausal when diagnosed with breast cancer, women with stage I breast cancer, women with breast cancer less than 2 years after surgery, and women with ER-negative breast cancer. Since no significant interaction was derived for each subgroup, there is a limit to concluding that there is a significant difference in the association for each subgroup. Considering that hs-CRP concentration tends to increase with age [39] and that hs-CRP concentrations are associated with increased risk of breast cancer recurrence and mortality [11,40], the result that hs-CRP concentrations decreased as the overall adherence score increased in these subgroups, including women in their 50s and older, and women who were postmenopausal when diagnosed with breast cancer, provides a meaningful basis for the importance of adhering to overall lifestyle guidelines in older breast cancer survivors. For adiponectin, a significant positive association with the overall adherence score was observed among all participants. However, as the sample size decreased in the subgroup analysis, only a few subgroups showed significant relationships regarding the tendency for adiponectin concentrations to increase with an increase in the overall adherence score. Lastly, IL-8 concentrations showed a significant decrease with increasing adherence scores only in the subgroup of participants under 50 years of age. However, this phenomenon cannot be generalized to all groups as the interaction by age group showed no significant difference, and there was no significant association in all participants and other subgroups. Further studies, such as clinical trials, are required to explore the association between overall lifestyle factors, including weight, physical activity, and diet, and inflammatory biomarkers in breast cancer survivors.

As far as we know, this is the first study to examine the association of lifestyle factors, assessed by adherence to the ACS guidelines on nutrition and physical activity for cancer survivors, with plasma levels of inflammatory markers among breast cancer survivors in Korea. In addition, this study included comprehensive information on a wide range of potential covariates using a structured questionnaire. However, this study also had some limitations that should be taken into consideration. First, this study was cross-sectional, so we could not infer causal relations between adherence to guidelines for cancer survivors and levels of inflammatory markers. Additionally, the sample size of this cross-sectional study was relatively small, which limited the ability to detect significant associations. To confirm the findings and explore the causal relationship between the overall lifestyle factors and inflammatory markers among breast cancer survivors, future research with a larger sample size and a longitudinal design is required. Second, dietary data were collected from 3DRs and FFQs. However, the overall acceptable agreement between the 2 measures [22] suggests that the ranking based on the guidelines may not differ according to the measure used. Self-reported dietary data assessed using either 3DRs or FFQ may be subject to measurement error, which is inherent in most dietary assessment methods [41,42]. In addition, dietary data assessed using FFQ has a limitation in that it is challenging to investigate the dietary habits that have recently gained attention, such as the consumption of highly processed foods, as the questionnaire is already limited to a predetermined list of foods for investigation. Third, non-fasting blood samples were used in this study; however, several studies have reported that biomarkers were highly correlated between non-fasting and fasting samples [43,44]. Fourth, inflammatory markers were measured at 2 different time points. To ensure comparability of the distributions, we used the residual method to account for variations in the measurement time point of the inflammatory markers. Lastly, although the participants in this study were from all around the country, these findings may not be generalizable to all breast cancer survivors in Korea.

In conclusion, adherence to lifestyle guidelines for cancer survivors is associated with beneficial effects on inflammatory marker levels, especially hs-CRP and adiponectin, among breast cancer survivors. Among the 3 components of the lifestyle guidelines—namely, BMI, physical activity, and diet—the BMI component exhibited the most similar tendency to the total score in relation to inflammatory indicators, while the diet component showed a significant association only with adiponectin. These findings did not vary by age at enrollment, menopausal status at diagnosis, AJCC breast cancer stage, time since surgery, or ER status. Further prospective and intervention studies are needed to investigate the relationship between adherence to the ACS guidelines on nutrition and physical activity for cancer survivors and the levels of inflammatory markers among breast cancer survivors.

## Figures and Tables

**Figure f1-epih-46-e2024026:**
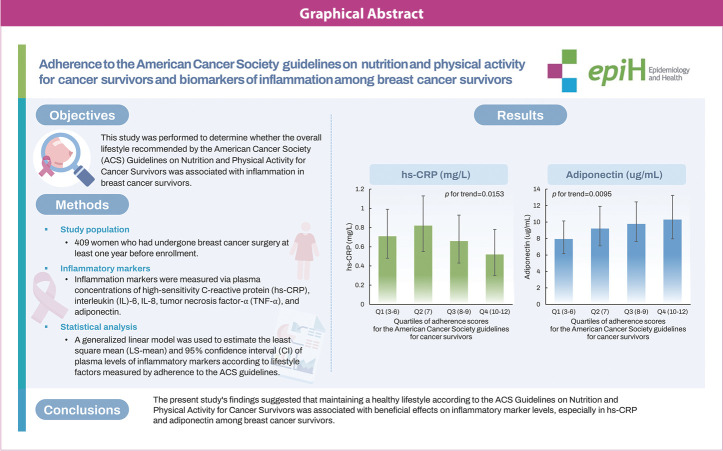


**Table 1.** Characteristics of study participants according to the adherence scores for the American Cancer Society guidelines for cancer survivors among breast cancer survivors (n=409)

Characteristics	Adherence score quartiles^1^				
	All (n=409)	Q1 (n=102)	Q2 (n=67)	Q3 (n=136)	Q4 (n=104)
Age (yr)	52.1±8.2	51.0±9.3	51.8±8.6	52.5±7.9	52.8±7.0
Body mass index (kg/m^2^)	23.4±3.1	24.9±4.2	24.1±2.7	22.9±2.6	22.1±1.9
Physical activity (MET-hr/wk)	33.3±38.7	12.1±10.8	16.1±14.7	30.4±33.3	68.9±47.9
Energy intake (kcal/day)	1,762.8±567.5	1,628.1±607.6	1,725.4±581.6	1,766.7±499.0	1,913.9±573.1
Education level					
Elementary school or below	52 (12.7)	18 (17.6)	9 (13.4)	13 (9.6)	12 (11.5)
Middle school	49 (12.0)	13 (12.7)	7 (10.4)	15 (11.0)	14 (13.5)
High school	201 (49.1)	51 (50.0)	34 (50.7)	66 (48.5)	50 (48.1)
College or above	107 (26.2)	20 (19.6)	17 (25.4)	42 (30.9)	28 (26.9)
Marital status					
Married or cohabiting	322 (78.7)	84 (82.4)	50 (74.6)	113 (83.1)	75 (72.1)
Unmarried, divorced or widowed	87 (21.3)	18 (17.6)	17 (25.4)	23 (16.9)	29 (27.9)
Menopausal status at diagnosis					
Premenopausal	268 (65.5)	62 (60.8)	48 (71.6)	88 (64.7)	70 (67.3)
Postmenopausal	141 (34.5)	40 (39.2)	19 (28.4)	48 (35.3)	34 (32.7)
AJCC stage at diagnosis					
I	200 (48.9)	52 (51.0)	26 (38.8)	72 (52.9)	50 (48.1)
II	162 (39.6)	44 (43.1)	27 (40.3)	50 (36.8)	41 (39.4)
III	47 (11.5)	6 (5.9)	14 (20.9)	14 (10.3)	13 (12.5)
Time since surgery (yr)					
1-<2	178 (43.5)	45 (44.1)	30 (44.8)	54 (39.7)	49 (47.1)
2-<5	150 (36.7)	36 (35.3)	23 (34.3)	53 (39.0)	38 (36.5)
≥5	81 (19.8)	21 (20.6)	14 (20.9)	29 (21.3)	17 (16.3)
ER status					
Negative	91 (22.3)	22 (21.6)	11 (16.4)	38 (27.9)	20 (19.2)
Positive	318 (77.8)	80 (78.4)	56 (83.6)	98 (72.1)	84 (80.8)
PR status					
Negative	129 (31.5)	29 (28.4)	19 (28.4)	49 (36.0)	32 (30.8)
Positive	280 (68.5)	73 (71.6)	48 (71.6)	87 (64.0)	72 (69.2)
History of chronic diseases					
No	324 (79.2)	77 (75.5)	54 (80.6)	108 (79.4)	85 (81.7)
Yes	85 (20.8)	25 (24.5)	13 (19.4)	28 (20.6)	19 (18.3)
Smoking status					
Never	376 (91.9)	88 (86.3)	61 (91.0)	128 (94.1)	99 (95.2)
Ever	33 (8.1)	14 (13.7)	6 (9.0)	8 (5.9)	5 (4.8)
Alcohol consumption (drink/day)					
Non-drinker	327 (80.0)	78 (76.5)	50 (74.6)	109 (80.1)	90 (86.5)
<1	34 (8.3)	8 (7.8)	5 (7.5)	11 (8.1)	10 (9.6)
≥1	48 (11.7)	16 (15.7)	12 (17.9)	16 (11.8)	4 (3.8)
Dietary supplement use					
No	146 (35.7)	45 (44.1)	32 (47.8)	46 (33.8)	23 (22.1)
Yes	263 (64.3)	57 (55.9)	35 (52.2)	90 (66.2)	81 (77.9)

Values are presented as mean±standard deviation or number (%). MET, metabolic equivalent task; AJCC, American Joint Committee on Cancer; ER, estrogen receptor; PR, progesterone receptor.

1 The American Cancer Society guidelines on nutrition and physical activity for cancer survivors score ranges from 3 to 12; When divided into quartiles, the score range for each quartile is as follows: Q1 (3-6), Q2 (7), Q3 (8-9), Q4 (10-12).

**Table 2.** Plasma levels of inflammatory markers according to the quartiles of adherence scores for the American Cancer Society guidelines for cancer survivors among breast cancer survivors (n=409)^1^

Variables	Plasma levels of inflammatory markers according to the adherence scores^2^				p for trend
	Q1	Q2	Q3	Q4	
hs-CRP (mg/L)	0.71 (0.48, 0.99)	0.82 (0.55, 1.13)	0.66 (0.43, 0.93)	0.52 (0.30, 0.78)	0.015
IL-6 (pg/mL)	0.89 (0.69, 1.12)	0.90 (0.68, 1.14)	0.81 (0.61, 1.03)	0.83 (0.63, 1.07)	0.405
IL-8 (pg/mL)	10.91 (7.70, 15.29)	10.09 (6.96, 14.44)	9.72 (6.82, 13.71)	9.53 (6.58, 13.63)	0.335
TNF-α (pg/mL)	10.55 (8.30, 13.35)	13.25 (10.33, 16.92)	12.11 (9.54, 15.32)	11.38 (8.86, 14.55)	0.759
Adiponectin (μg/mL)	7.94 (6.17, 10.14)	9.22 (7.10, 11.91)	9.77 (7.62, 12.45)	10.30 (7.96, 13.24)	0.009

Values are presented as least square mean (95% confidence interval). hs-CRP, high-sensitivity C-reactive protein; IL-6, interleukin-6; IL-8, interleukin-8; TNF-α, tumor necrosis factor-α.

1 Models were adjusted for age (years; continuous), energy intake (log-transformed energy intake, kcal/day; continuous), education level (elementary school or below, middle school, high school, or college or above), marital status (married or cohabiting, unmarried or divorced or widowed), menopausal status at diagnosis (premenopausal or postmenopausal), stage (I, II, or III), time since surgery (1 to <2, 2 to <5, or ≥5 years), estrogen receptor status (negative, positive), history of chronic disease (yes or no), smoking status (never or ever), alcohol intake (non-drinker, <1, ≥1 drink/day), dietary supplement use (yes or no), and medical center (5 centers).

2 The American Cancer Society guidelines on nutrition and physical activity for cancer survivors score ranges from 3 to 12; When divided into quartiles, the score range for each quartile is as follows: Q1 (3-6), Q2 (7), Q3 (8-9), Q4 (10-12).

**Table 3.** Plasma levels of inflammatory markers according to the scores for body mass index, physical activity, and diet in the American Cancer Society guidelines for cancer survivors among breast cancer survivors (n=409)^1^

Variables	Plasma levels of inflammatory markers according to the adherence scores				p for trend
	Score 1	Score 2	Score 3	Score 4	
Body mass index (kg/m^2^)	<18.5 or ≥30.0	25.0-<30.0	23.0-<25.0	18.5-<23.0	
hs-CRP (mg/L)	0.84 (0.51, 1.23)	0.84 (0.58, 1.14)	0.65 (0.42, 0.93)	0.60 (0.39, 0.84)	0.004
IL-6 (pg/mL)	0.90 (0.64, 1.21)	0.91 (0.71, 1.15)	0.80 (0.60, 1.03)	0.85 (0.66, 1.07)	0.434
IL-8 (pg/mL)	11.68 (7.43, 18.05)	11.14 (7.85, 15.67)	10.31 (7.19, 14.64)	9.30 (6.64, 12.89)	0.070
TNF-α (pg/mL)	13.87 (10.20, 18.73)	12.26 (9.64, 15.52)	10.41 (8.12, 13.29)	11.61 (9.25, 14.52)	0.217
Adiponectin (μg/mL)	9.77 (7.10, 13.31)	7.81 (6.06, 10.00)	8.09 (6.25, 10.39)	10.33 (8.20, 12.97)	0.010
Physical activity (MET-hr/wk)	<11.7	11.7-24.5	24.6-46.6	≥46.9	
hs-CRP (mg/L)	0.74 (0.51, 1.02)	0.62 (0.39, 0.90)	0.71 (0.45, 1.00)	0.62 (0.39, 0.90)	0.333
IL-6 (pg/mL)	0.91 (0.71, 1.13)	0.82 (0.62, 1.05)	0.83 (0.62, 1.07)	0.81 (0.60, 1.03)	0.219
IL-8 (pg/mL)	10.26 (7.33, 14.22)	10.31 (7.15, 14.69)	9.83 (6.75, 14.13)	10.11 (7.01, 14.41)	0.825
TNF-α (pg/mL)	10.87 (8.63, 13.63)	12.69 (9.91, 16.18)	12.66 (9.83, 16.22)	12.20 (9.52, 15.56)	0.211
Adiponectin (μg/mL)	8.48 (6.66, 10.74)	9.69 (7.48, 12.47)	9.79 (7.51, 12.66)	9.43 (7.28, 12.15)	0.255
Diet^2^					
hs-CRP (mg/L)	0.70 (0.45, 0.98)	0.74 (0.50, 1.02)	0.69 (0.46, 0.96)	0.56 (0.31, 0.84)	0.226
IL-6 (pg/mL)	0.86 (0.65, 1.10)	0.85 (0.65, 1.07)	0.87 (0.67, 1.10)	0.89 (0.66, 1.15)	0.737
IL-8 (pg/mL)	10.58 (7.36, 15.04)	10.05 (7.08, 14.10)	9.72 (6.85, 13.64)	10.91 (7.35, 15.99)	0.976
TNF-α (pg/mL)	11.28 (8.78, 14.40)	11.60 (9.13, 14.67)	12.22 (9.65, 15.43)	11.48 (8.75, 14.99)	0.678
Adiponectin (μg/mL)	8.32 (6.41, 10.73)	8.87 (6.92, 11.31)	9.43 (7.37, 11.99)	10.97 (8.32, 14.38)	0.020

Values are presented as least square mean (95% confidence interval). hs-CRP, high-sensitivity C-reactive protein; IL-6, interleukin-6; IL-8, interleukin-8; TNF-α, tumor necrosis factor-α.

1 Models were adjusted for age (years; continuous), energy intake (log-transformed energy intake, kcal/day; continuous), education level (elementary school or below, middle school, high school, or college or above), marital status (married or cohabiting, unmarried or divorced or widowed), menopausal status at diagnosis (premenopausal or postmenopausal), stage (I, II, or III), time since surgery (1 to <2, 2 to <5, or ≥5 years), estrogen receptor status (negative, positive), history of chronic disease (yes or no), smoking status (never or ever), alcohol intake (non-drinker, <1, ≥1 drink/day), dietary supplement use (yes or no), and medical center (5 centers).

2 Diet score was calculated based on the intake of 3 food groups: fruits and vegetables, whole grains, and red and processed meat; More specifically, diet score was calculated based on the amount of fruit and vegetable intake, with 1 being the lowest quartile (<362.5 g) and 4 being the highest quartile (>833.0 g); Similarly, the score was calculated based on the amount of whole grain intake with 1 being the lowest quartile (<44.1 g) and 4 being the highest quartile (>150 g); For, red and processed meat, the lowest quartile (<12.3 g) received the highest score, and the highest quartile (>90.4 g) received the lowest score; Finally, the scores of the 3 food groups were summed and classified into 4 groups based on the quartiles of the calculated score (scored 1 [lowest quartile] to 4 [highest quartile]); Median values for each score of diet component are as follows (a score of 1: 265.0 g of fruits and vegetables, 13.6 g of whole grains, 91.8 g of red and processed meat), (a score of 2: 453.6 g of fruits and vegetables, 73.4 g of whole grains, 47.9 g of red and processed meat), (a score of 3: 625.8 g of fruits and vegetables, 140.7 g of whole grains, 35.2 g of red and processed meat), and (a score of 4: 859.1 g of fruits and vegetables, 154.0 g of whole grains, 10.0 g of red and processed meat).

**Table 4.** Plasma levels of inflammatory markers according to the quartiles of adherence scores for the American Cancer Society guidelines for cancer survivors by age at enrollment, menopausal status at diagnosis, AJCC stage at diagnosis, time since surgery, and ER status among breast cancer survivors (n=409)^1^

Variables	n	Plasma levels of inflammatory markers according to the adherence scores^2^				p for trend	p for interaction
		Q1	Q2	Q3	Q4		
hs-CRP (mg/L)							
Age at enrollment (yr)							0.219
<50	162	0.58 (0.15, 1.16)	0.65 (0.18, 1.31)	0.54 (0.11, 1.13)	0.49 (0.07, 1.07)	0.405	
≥50	247	0.92 (0.55, 1.39)	1.02 (0.61, 1.54)	0.77 (0.44, 1.19)	0.54 (0.24, 0.92)	0.002	
Menopausal status at diagnosis							0.542
Premenopausal	268	0.63 (0.33, 0.99)	0.60 (0.30, 0.98)	0.62 (0.32, 0.99)	0.46 (0.18, 0.80)	0.153	
Postmenopausal	141	0.77 (0.35, 1.32)	1.32 (0.78, 2.04)	0.62 (0.25, 1.09)	0.52 (0.16, 0.99)	0.027	
AJCC stage at diagnosis							0.795
Stage I	200	0.84 (0.48, 1.30)	0.86 (0.44, 1.42)	0.63 (0.30, 1.05)	0.56 (0.23, 0.98)	0.018	
Stage II-III	209	0.63 (0.29, 1.07)	0.79 (0.43, 1.26)	0.68 (0.32, 1.13)	0.47 (0.16, 0.87)	0.134	
Time since surgery (yr)							0.481
1-<2	178	0.50 (0.21, 0.87)	0.61 (0.27, 1.06)	0.34 (0.06, 0.69)	0.26 (-0.01, 0.59)	0.011	
≥2	231	0.95 (0.53, 1.49)	1.03 (0.60, 1.57)	0.99 (0.56, 1.52)	0.82 (0.42, 1.34)	0.329	
ER status							0.377
Negative	91	0.78 (0.27, 1.50)	0.54 (-0.02, 1.40)	0.46 (0.03, 1.07)	0.19 (-0.18, 0.71)	0.019	
Positive	318	0.69 (0.44, 0.97)	0.84 (0.56, 1.17)	0.65 (0.41, 0.94)	0.58 (0.34, 0.86)	0.130	
IL-6 (pg/mL)							
Age at enrollment (yr)							0.543
<50	162	0.63 (0.30, 1.03)	0.71 (0.35, 1.17)	0.61 (0.28, 1.03)	0.51 (0.20, 0.91)	0.156	
≥50	247	0.96 (0.65, 1.33)	0.93 (0.61, 1.31)	0.86 (0.57, 1.20)	0.96 (0.65, 1.34)	0.938	
Menopausal status at diagnosis							0.972
Premenopausal	268	0.81 (0.56, 1.10)	0.79 (0.53, 1.09)	0.72 (0.48, 1.00)	0.75 (0.50, 1.05)	0.559	
Postmenopausal	141	1.01 (0.60, 1.51)	1.06 (0.64, 1.58)	0.89 (0.53, 1.35)	0.87 (0.49, 1.35)	0.303	
AJCC stage at diagnosis							0.884
Stage I	200	0.94 (0.63, 1.32)	1.08 (0.69, 1.56)	0.91 (0.59, 1.29)	0.89 (0.56, 1.29)	0.452	
Stage II-III	209	0.79 (0.51, 1.12)	0.75 (0.48, 1.06)	0.70 (0.43, 1.01)	0.72 (0.44, 1.04)	0.541	
Time since surgery (yr)							0.984
1-<2	178	0.93 (0.61, 1.32)	0.86 (0.52, 1.28)	0.72 (0.41, 1.10)	0.78 (0.46, 1.16)	0.231	
≥2	231	0.88 (0.60, 1.22)	0.99 (0.70, 1.35)	0.91 (0.63, 1.25)	0.91 (0.61, 1.26)	0.988	
ER status							0.360
Negative	91	0.94 (0.45, 1.59)	0.90 (0.30, 1.77)	1.04 (0.51, 1.74)	1.19 (0.61, 1.99)	0.364	
Positive	318	0.87 (0.67, 1.10)	0.88 (0.67, 1.12)	0.78 (0.58, 0.99)	0.79 (0.59, 1.02)	0.270	
IL-8 (pg/mL)							
Age at enrollment (yr)							0.114
<50	162	11.72 (5.05, 25.74)	11.05 (4.43, 25.73)	8.45 (3.35, 19.53)	7.48 (2.87, 17.56)	0.037	
≥50	247	11.85 (7.42, 18.61)	10.49 (6.39, 16.85)	11.79 (7.51, 18.22)	12.41 (7.77, 19.50)	0.628	
Menopausal status at diagnosis							0.737
Premenopausal	268	11.93 (7.59, 18.44)	11.40 (7.11, 17.97)	10.15 (6.34, 15.92)	11.17 (6.95, 17.63)	0.719	
Postmenopausal	141	9.47 (4.53, 18.82)	8.31 (3.93, 16.59)	10.34 (5.18, 19.81)	7.85 (3.67, 15.77)	0.492	
AJCC stage at diagnosis							0.933
Stage I	200	9.86 (5.78, 16.39)	8.76 (4.61, 15.97)	9.32 (5.34, 15.80)	7.68 (4.21, 13.45)	0.199	
Stage II-III	209	10.32 (6.02, 17.26)	8.67 (5.06, 14.45)	7.23 (4.07, 12.35)	8.54 (4.83, 14.62)	0.419	
Time since surgery (yr)							0.250
1-<2	178	13.20 (7.52, 22.67)	7.63 (3.88, 14.25)	8.32 (4.37, 15.16)	8.54 (4.50, 15.55)	0.138	
≥2	231	11.47 (6.91, 18.66)	13.29 (8.12, 21.37)	12.39 (7.55, 19.96)	11.82 (7.01, 19.52)	0.986	
ER status							0.973
Negative	91	10.95 (4.54, 24.81)	8.34 (2.41, 24.60)	12.22 (5.00, 28.10)	12.90 (5.09, 30.74)	0.650	
Positive	318	10.05 (7.00, 14.27)	9.55 (6.54, 13.78)	8.57 (5.90, 12.28)	8.47 (5.77, 12.26)	0.218	
TNF-α (pg/mL)							
Age at enrollment (yr)							0.318
<50	162	8.89 (5.04, 15.18)	9.95 (5.45, 17.57)	7.71 (4.21, 13.57)	7.49 (4.05, 13.27)	0.154	
≥50	247	11.22 (8.13, 15.34)	14.21 (10.23, 19.60)	13.92 (10.27, 18.75)	13.00 (9.46, 17.75)	0.389	
Menopausal status at diagnosis							0.526
Premenopausal	268	10.46 (7.67, 14.15)	12.64 (9.20, 17.25)	12.64 (9.25, 17.15)	10.75 (7.78, 14.73)	0.852	
Postmenopausal	141	10.93 (6.62, 17.67)	14.49 (8.92, 23.20)	11.97 (7.47, 18.86)	13.64 (8.35, 21.92)	0.259	
AJCC stage at diagnosis							0.464
Stage I	200	10.84 (7.21, 16.07)	12.81 (7.98, 20.24)	13.44 (8.89, 20.09)	12.49 (8.07, 19.05)	0.375	
Stage II-III	209	9.81 (6.99, 13.63)	12.88 (9.32, 17.65)	10.71 (7.62, 14.90)	10.11 (7.14, 14.17)	0.668	
Time since surgery (yr)							0.651
1-<2	178	8.09 (5.78, 11.18)	9.96 (6.91, 14.20)	9.35 (6.54, 13.19)	8.48 (5.91, 12.01)	0.985	
≥2	231	10.90 (7.25, 16.19)	14.19 (9.58, 20.81)	12.22 (8.21, 17.97)	11.81 (7.77, 17.72)	0.833	
ER status							0.988
Negative	91	10.48 (6.40, 16.82)	8.99 (4.62, 16.76)	14.40 (8.81, 23.16)	11.64 (6.89, 19.25)	0.767	
Positive	318	10.67 (8.22, 13.76)	14.01 (10.75, 18.16)	11.97 (9.22, 15.45)	11.64 (8.90, 15.15)	0.809	
Adiponectin (μg/mL)							
Age at enrollment (yr)							0.818
<50	162	12.04 (6.68, 21.13)	16.50 (8.92, 29.86)	16.14 (8.87, 28.77)	16.66 (9.11, 29.85)	0.070	
≥50	247	8.17 (5.79, 11.37)	8.68 (6.08, 12.23)	9.33 (6.73, 12.80)	9.76 (6.96, 13.55)	0.147	
Menopausal status at diagnosis							0.602
Premenopausal	268	7.65 (5.47, 10.56)	9.05 (6.43, 12.59)	9.88 (7.08, 13.63)	10.30 (7.35, 14.30)	0.028	
Postmenopausal	141	7.34 (4.43, 11.81)	8.71 (5.33, 13.89)	7.72 (4.80, 12.12)	8.15 (4.96, 13.06)	0.618	
AJCC stage at diagnosis							0.989
Stage I	200	7.82 (5.22, 11.52)	8.76 (5.47, 13.74)	9.51 (6.31, 14.11)	10.01 (6.53, 15.09)	0.074	
Stage II-III	209	6.81 (4.69, 9.73)	8.51 (5.97, 11.98)	8.75 (6.07, 12.44)	10.03 (6.95, 14.29)	0.013	
Time since surgery (yr)							0.712
1-<2	178	9.47 (6.46, 13.70)	11.10 (7.29, 16.67)	12.93 (8.66, 19.08)	12.36 (8.26, 18.26)	0.079	
≥2	231	8.95 (5.99, 13.17)	9.94 (6.73, 14.50)	10.61 (7.20, 15.45)	11.68 (7.80, 17.26)	0.044	
ER status							0.291
Negative	91	6.67 (3.98, 10.82)	7.41 (3.78, 13.79)	7.45 (4.43, 12.15)	9.89 (5.85, 16.30)	0.093	
Positive	318	8.67 (6.61, 11.30)	10.05 (7.61, 13.19)	10.99 (8.40, 14.29)	10.77 (8.17, 14.11)	0.056	

Values are presented as least square mean (95% confidence interval). AJCC stage, American Joint Committee on Cancer stage; ER status, estrogen receptor status; hs-CRP, high-sensitivity C-reactive protein; IL-6, interleukin-6; IL-8, interleukin-8; TNF-α, tumor necrosis factor-α.

1 Models were adjusted for age (years; continuous), energy intake (log-transformed energy intake, kcal/day; continuous), education level (elementary school or below, middle school, high school, or college or above), marital status (married or cohabiting, unmarried or divorced or widowed), menopausal status at diagnosis (premenopausal or postmenopausal), stage (I, II, or III), time since surgery (1 to <2, 2 to <5, or ≥5 years), estrogen receptor status (negative, positive), history of chronic disease (yes or no), smoking status (never or ever), alcohol intake (non-drinker, <1, ≥1 drink/day), dietary supplement use (yes or no), and medical center (5 centers).

2 The American Cancer Society guidelines on nutrition and physical activity for cancer survivors score ranges from 3 to 12; When divided into quartiles, the score range for each quartile is as follows: Q1 (3-6), Q2 (7), Q3 (8-9), Q4 (10-12).

## Supplementary materials

Supplementary Material 1. Least square means (LS-means) and 95% confidence intervals (CIs) of plasma levels of inflammatory markers according to the quartiles of adherence scores for the American Cancer Society guidelines for cancer survivors among breast cancer survivors after excluding participants with replacement values

Supplementary Material 2. Least square means (LS-means) and 95% confidence intervals (CIs) of plasma levels of inflammatory markers according to the scores for body mass index, physical activity, and diet in the American Cancer Society guidelines for cancer survivors among breast cancer survivors (n = 409)
