# Supplementary material for: Adherence to the American Cancer Society guidelines on nutrition and physical activity for cancer survivors and biomarkers of inflammation among breast cancer survivors
Source: Epidemiol Health. 2024 Jan 25;46:e2024026. doi: 10.4178/epih.e2024026 (PMC11099571; doi:10.4178/epih.e2024026)
Supplement: Supplementary Material 1. — Least square means (LS-means) and 95% confidence intervals (CIs) of plasma levels of inflammatory markers according to the quartiles of adherence scores for the American Cancer Society guidelines for cancer survivors among breast cancer survivors after excluding participants with replacement values [file epih-46-e2024026-Supplementary-1.docx]

Supplementary material 1. Least square means (LS-means) and 95% confidence intervals (CIs) of plasma levels of inflammatory markers according to the quartiles of adherence scores for the American Cancer Society guidelines for cancer survivors among breast cancer survivors after excluding participants with replacement values^1),2)^

|  |  | LS-Means (95% CIs) of plasma levels of inflammatory markers according to the adherence scores | | | | p for trend |
| --- | --- | --- | --- | --- | --- | --- |
|  | Sample size after excluding participants with replacement value | Q1 | Q2 | Q3 | Q4 |  |
| hs-CRP (mg/L) | 342 (83.6%) | 0.84 (0.57-1.16) | 0.96 (0.66-1.31) | 0.81 (0.54-1.13) | 0.62 (0.37-0.92) | 0.0203 |
| IL-6 (pg/mL) | 387 (94.6%) | 0.90 (0.70-1.12) | 0.96 (0.74-1.20) | 0.86 (0.66-1.08) | 0.88 (0.68-1.11) | 0.6534 |
| IL-8 (pg/mL) | 409 (100%) | 10.91 (7.70-15.29) | 10.09 (6.96-14.44) | 9.72 (6.82-13.71) | 9.53 (6.58-13.63) | 0.3353 |
| TNF-a (pg/mL) | 398 (97.3%) | 12.46 (10.14-15.26) | 14.87 (12.03-18.34) | 13.50 (11.00-16.51) | 12.56 (10.14-15.51) | 0.6551 |
| Adiponectin (ug/mL) | 409 (100%) | 7.94 (6.17-10.14) | 9.22 (7.10-11.91) | 9.77 (7.62-12.45) | 10.30 (7.96-13.24) | 0.0095 |

^1)^ The American Cancer Society guidelines on nutrition and physical activity for cancer survivors score ranges from 3 to 12. When divided into quartiles, the score range for each quartile is as follows: Q1 (3-6), Q2 (7), Q3 (8-9), Q4 (10-12). ^2)^ Models were adjusted for age (years; continuous), energy intake (log-transformed energy intake, kcal/day; continuous), education level (elementary school or below, middle school, high school, or college or above), marital status (married or cohabiting, unmarried or divorced or widowed), menopausal status at diagnosis (premenopausal or postmenopausal), stage (I, II, or III), time since surgery (1 to < 2 years, 2 to < 5 years, or ≥ 5 years), estrogen receptor status (negative, positive), history of chronic disease (yes or no), smoking status (never or ever), alcohol intake (non-drinker, < 1 cup/day, ≥ 1 cup/day), dietary supplement use (yes or no), and medical center (five centers).
