# Supplementary material for: Adherence to the American Cancer Society guidelines on nutrition and physical activity for cancer survivors and biomarkers of inflammation among breast cancer survivors
Source: Epidemiol Health. 2024 Jan 25;46:e2024026. doi: 10.4178/epih.e2024026 (PMC11099571; doi:10.4178/epih.e2024026)
Supplement: Supplementary Material 2. — Least square means (LS-means) and 95% confidence intervals (CIs) of plasma levels of inflammatory markers according to the scores for body mass index, physical activity, and diet in the American Cancer Society guidelines for cancer survivors among breast cancer survivors (n = 409) [file epih-46-e2024026-Supplementary-2.docx]

Supplementary material 2. Least square means (LS-means) and 95% confidence intervals (CIs) of plasma levels of inflammatory markers according to the scores for body mass index, physical activity, and diet in the American Cancer Society guidelines for cancer survivors among breast cancer survivors (n = 409)^1)^

|  | LS-Means (95% CIs) of plasma levels of inflammatory markers according to the adherence scores | | | | p for trend |
| --- | --- | --- | --- | --- | --- |
| **Body mass index** | Score 1  (<18.5 or ≥30 kg/m^2^) | Score 2  (25-< 30 kg/m^2^) | Score 3  (23-<25 kg/m^2^) | Score 4  (18.5-<23 kg/m^2^) |  |
| hs-CRP (mg/L) | 0.79 (0.47-1.17) | 0.79 (0.54-1.09) | 0.62 (0.38-0.89) | 0.56 (0.35-0.81) | 0.0057 |
| IL-6 (pg/mL) | 0.90 (0.63-1.21) | 0.89 (0.68-1.13) | 0.79 (0.59-1.02) | 0.84 (0.65-1.06) | 0.4960 |
| IL-8 (pg/mL) | 11.87 (7.49-18.50) | 11.24 (7.84-15.95) | 10.42 (7.21-14.89) | 9.41 (6.66-13.14) | 0.0750 |
| TNF-a (pg/mL) | 14.22 (10.42-19.28) | 12.79 (10.01-16.26) | 10.69 (8.30-13.68) | 11.93 (9.46-14.97) | 0.1749 |
| Adiponectin (ug/mL) | 10.49 (7.62-14.31) | 8.29 (6.42-10.63) | 8.57 (6.61-11.02) | 10.96 (8.68-13.78) | 0.0123 |
| **Physical Activity** | Score 1  (<11.7 MET-hours/week) | Score 2  (11.7–24.5 MET-hours/week) | Score 3  (24.6–46.6 MET-hours/week) | Score 4  (≥46.9 MET-hours/week) |  |
| hs-CRP (mg/L) | 0.74 (0.50-1.02) | 0.63 (0.39-0.91) | 0.71 (0.46-1.01) | 0.67 (0.43-0.96) | 0.6891 |
| IL-6 (pg/mL) | 0.92 (0.71-1.15) | 0.84 (0.62-1.08) | 0.84 (0.63-1.09) | 0.82 (0.61-1.05) | 0.2472 |
| IL-8 (pg/mL) | 10.75 (7.60-15.05) | 10.98 (7.55-15.78) | 10.26 (7.00-14.85) | 10.81 (7.44-15.53) | 0.9046 |
| TNF-a (pg/mL) | 11.04 (8.71-13.94) | 12.88 (10.00-16.51) | 12.97 (10.03-16.69) | 12.57 (9.76-16.12) | 0.1544 |
| Adiponectin (ug/mL) | 8.86 (6.94-11.23) | 9.92 (7.65-12.79) | 10.18 (7.83-13.16) | 9.14 (7.04-11.79) | 0.6422 |
| **Diet** | Score 1^2)^ | Score 2^2)^ | Score 3^2)^ | Score 4^2)^ |  |
| hs-CRP (mg/L) | 0.70 (0.45-0.99) | 0.76 (0.51-1.05) | 0.72 (0.48-1.01) | 0.58 (0.32-0.88) | 0.3159 |
| IL-6 (pg/mL) | 0.85 (0.64-1.09) | 0.83 (0.63-1.06) | 0.86 (0.66-1.09) | 0.88 (0.64-1.15) | 0.6524 |
| IL-8 (pg/mL) | 10.87 (7.49-15.58) | 10.44 (7.29-14.81) | 10.14 (7.08-14.37) | 11.37 (7.57-16.86) | 0.9109 |
| TNF-a (pg/mL) | 12.07 (9.37-15.46) | 12.27 (9.62-15.59) | 12.89 (10.12-16.35) | 12.16 (9.21-15.96) | 0.7519 |
| Adiponectin (ug/mL) | 8.60 (6.62-11.09) | 8.90 (6.92-11.38) | 9.46 (7.38-12.07) | 11.28 (8.52-14.82) | 0.0233 |

^1)^ Models were adjusted for age (years; continuous), energy intake (log-transformed energy intake, kcal/day; continuous), education level (elementary school or below, middle school, high school, or college or above), marital status (married or cohabiting, unmarried or divorced or widowed), menopausal status at diagnosis (premenopausal or postmenopausal), stage (I, II, or III), time since surgery (1 to < 2 years, 2 to < 5 years, or ≥ 5 years), estrogen receptor status (negative, positive), history of chronic disease (yes or no), smoking status (never or ever), alcohol intake (non-drinker, < 1 cup/day, ≥ 1 cup/day), dietary supplement use (yes or no), and medical center (five centers). In each component analysis (i.e., body mass index, physical activity, diet), two other components were additionally adjusted in addition to the aforementioned covariates. ^2)^ Diet score was calculated based on the intake of three food groups: fruits and vegetables, whole grains, and red and processed meat. More specifically, diet score was calculated based on the amount of fruit and vegetable intake, with 1 being the lowest quartile (< 362.5 g) and 4 being the highest quartile (> 833.0 g). Similarly, the score was calculated based on the amount of whole grain intake with 1 being the lowest quartile (< 44.1 g) and 4 being the highest quartile (> 150 g). For, red and processed meat, the lowest quartile (<12.3 g) received the highest score, and the highest quartile (>90.4 g) received the lowest score. Finally, The scores of the three food groups were summed and classified into four groups based on the quartiles of the calculated score [scored 1 (lowest quartile) to 4 (highest quartile)]. Median values for each score of diet component are as follows (Score 1: 265.0 g of fruits and vegetables, 13.6 g of whole grain, 91.8 g of red and processed meat), (Score 2: 453.6 g of fruits and vegetables, 73.4 g of whole grain, 47.9 g of red and processed meat), (Score 3: 625.8 g of fruits and vegetables, 140.7 g of whole grain, 35.2 g of red and processed meat), and (Score 4: 859.1 g of fruits and vegetables, 154.0 g of whole grain, 10.0 g of red and processed meat).
